# Construction of Pancreatic Cancer Classifier Based on SVM Optimized by Improved FOA

**DOI:** 10.1155/2015/781023

**Published:** 2015-10-12

**Authors:** Huiyan Jiang, Di Zhao, Ruiping Zheng, Xiaoqi Ma

**Affiliations:** ^1^Software College, Northeastern University, Shenyang 110819, China; ^2^School of Science and Technology, Nottingham Trent University, Nottingham NG17 8NU, UK

## Abstract

A novel method is proposed to establish the pancreatic cancer classifier. Firstly, the concept of quantum and fruit fly optimal algorithm (FOA) are introduced, respectively. Then FOA is improved by quantum coding and quantum operation, and a new smell concentration determination function is defined. Finally, the improved FOA is used to optimize the parameters of support vector machine (SVM) and the classifier is established by optimized SVM. In order to verify the effectiveness of the proposed method, SVM and other classification methods have been chosen as the comparing methods. The experimental results show that the proposed method can improve the classifier performance and cost less time.

## 1. Introduction

Pancreatic cancer is one of the world's top 10 malignant tumors [1]. Its early and accurate diagnosis is difficult. Once the diagnosis is confirmed, the tumor has reached an advanced stage. It is of great significance to improve prognosis for early detection, early diagnosis, and early treatment [2]. With the development of computer science and computer image-processing technology, computer aided detection (CAD) technology is established. CAD systems are increasingly used as an aid by radiologists for detection and interpretation of diseases [3], reducing the burden of doctors and improving the diagnosis accuracy.

Image recognition is one of the most important parts of CAD technology. The recognition process is mainly divided into two phases, namely, feature extraction and selection and classifier construction. In [4], we argue that tensors can describe space information among image features and need less space than vectors. Multilinear principal component analysis (MPCA) method [5] can be used to select the core tensors. In this paper, we also use tensors to represent CT images and MPCA to select core tensors to reduce the tensor dimension.

There are many methods to establish the medical image classifier. Kovalerchuk et al. [6] and Pendharkar et al. [7] used machine learning and data mining technology in breast cancer detection. In recent years, many researchers have made thorough research on medical image classification. Antonie et al. [8] combined association rule and neural network to mine the texture feature in different regions of breast images and realized the automatic diagnosis of breast cancer. Zhang et al. [9] classified cervix uterus lymphonodus by support vector machine (SVM) and size and shape features. Ramírez et al. [10] proposed to use neural network method in classification of brain images of Alzheimer's disease.

However, the research of pancreatic cancer classification is in a fledging period. Tsai and Kojma [11] proposed the pancreatic tiny anomaly detection method for CT images and introduced the square of logarithm operation in grayscale to enhance the margin of low grayscale. Takada et al. [12] proposed a new pancreatic classification system to distinguish the four parts of pancreas based on the anatomy of pancreas and their own experience. He et al. [13] proposed a novel group search optimizer- (GSO-) based biomarker discovery method for pancreatic cancer diagnosis using mass spectrometry data, compared with a genetic algorithm, evolution strategies, evolutionary programming, and a particle swarm optimizer and achieved better classification performance than other algorithms.

Theoretically, imaging examination of any body tissues and organs can use CAD technology to improve diagnostic accuracy. However, since the position of pancreas is covert and has complex relationship with other organs, the pancreatic cancer image classification is difficult.

In this paper, we employ SVM [14], which are suitable for solving small-sample learning and nonlinear and high dimension problems, to establish the pancreatic cancer classification, and improve fruit fly optimization algorithm (FOA) [15] to optimize parameters of SVM. We provide a new fitness function which is more in line with the actual clinical needs in *K*-fold cross-validation to assess the classifier performance. Using the above strategies, the classification performance can be improved. Experimental results on pancreatic regions of abdominal CT images demonstrate the feasibility and efficiency of the proposed method.

This paper is organized as follows.  Section 2 introduces the background of this researching, including support vector machine, fruit fly optimization algorithm, and the concept of quantum.  Section 3 illustrates the method of construction of SVM classifier based on improved FOA.  Section 4 presents the experimental data and the evaluation criterions, showing the results of the pancreatic cancer classification based on the improved FOA and other comparative methods. It also discusses the experiment results.  Section 5 concludes the work in this paper.

## 2. Background

We introduce SVM and FOA in this part; the concept of quantum is shown in [4].

### 2.1. Support Vector Machine

Support vector machine (SVM) [14] is built on statistical learning theory. It is suitable for small-sample learning and nonlinear and high dimension problem. SVM is based on the principle of structural risk minimization and has strong generalization ability. It studies optimal separating hyperplane in the high dimension feature space for sample classification.

SVM mainly aims at binary classification. For linear separable problem, we consider samples as (*x*_*i*_, *y*_*i*_). *x*_*i*_ ∈ *R* ^*n*^ is the feature set of medical images, *y*_*i*_ ∈ {+1, −1} is the label of samples, and *i* = 1,…, *l*,  *l* is the number of samples. The optimal separating hyperplane is *f*(*x*) = *ω* ^*T*^ *x* + *b* = 0. The functional margin which is the distance from a sample point to separating hyperplane is ${\hat{\gamma }}_{i}=y_{i}\left(\omega^{T}x_{i}+b\right)=y_{i}f(x_{i})$ . The geometrical margin $\tilde{\gamma }=y\hat{\gamma }=\hat{\gamma }/\left\|\omega \right\|$ is obtained by normalizing *ω*, and it is simplified as $\tilde{\gamma }=1/\left\|\omega \right\|$ . The objective is to obtain the maximum value of $\tilde{\gamma }$ . It is equivalent to obtain the minimum value of ‖*ω*‖. Finally, the problem translates into the quadratic programming problem as in (1), where *C* is penalty coefficient, and *ξ*_*i*_ is slack variable.

The Lagrange duality translation is conducted for (1). And (1) translates into dual problem as (2):

(1) $$\begin{array}{c} \min\;\frac{1}{2}{\left\|\omega \right\|}^{2}+C\overset{l}{\underset{i=1}{\sum }}\xi_{i}\\ \text{s.t.}\;y_{i}\left(w^{T}x+b\right)\geq 1-\xi_{i},\;i=1,\ldots ,l \end{array}$$

(2) $$\begin{array}{c} \underset{\alpha }{\max}\;\overset{l}{\underset{i=1}{\sum }}\alpha_{i}-\frac{1}{2}\overset{l}{\underset{i,j=1}{\sum }}\alpha_{i}\alpha_{j}y_{i}y_{j}\langle x_{i},x_{j}\rangle \\ \text{s.t.}\;0\leq \alpha_{i}\leq C,\;i=1,\ldots ,l,\;\overset{l}{\underset{i=1}{\sum }}\alpha_{i}y_{i}=0. \end{array}$$

The optimal separating function is shown as

(3) $$\begin{array}{c} f\left(x\right)=\mathrm{sgn}\left(\overset{l}{\underset{i=1}{\sum }}\alpha_{i}y_{i}\langle x_{i},x\rangle +b\right). \end{array}$$

For nonlinear problem, the kernel function is used to translate nonlinear problem in low dimensional space into linear problem in high dimensional space. The optimal separating function is shown as

(4) $$\begin{array}{c} f\left(x\right)=\mathrm{sgn}\left(\overset{l}{\underset{i=1}{\sum }}\alpha_{i}y_{i}K\langle x_{i},x\rangle +b\right). \end{array}$$

The staple kernel functions are shown as (5)~(8). In this paper, the radial basis function (RBF) as (7) is used.

Linear kernel is

(5) $$\begin{array}{c} f\left(x\right)=x^{\prime }x. \end{array}$$

Polynomial kernel is

(6) $$\begin{array}{c} f\left(x\right)={\left(\gamma \cdot x^{\prime }\cdot x+b\right)}^{d}. \end{array}$$

RBF kernel is

(7) $$\begin{array}{c} f\left(x\right)=\exp\left(-\gamma \cdot {\left\|x-x^{\prime }\right\|}^{2}\right). \end{array}$$

Sigmoid kernel is

(8) $$\begin{array}{c} f\left(x\right)=\tanh\left(\gamma \cdot x^{\prime }\cdot x+b\right). \end{array}$$

The main influencing factor of recognition performance is the parameters used in SVM. Presently the staple methods to select optimal parameters include grid search [16], genetic algorithm (GA) [17], and particle swarm optimization (PSO) [18] algorithm. In [19], Dorigo et al. proposed ant colony optimization (ACO) algorithm to select optimal parameters value, achieving better classification performance while taking more time. In [20, 21], Xu et al. and Tiwari and Vidyarthi proposed quantum genetic algorithm (QGA) to optimize SVM parameters and verified that quantum operation can increase the scope of the search space and has good searching ability. In [4], Jiang et al. used quantum simulated annealing (QSA) algorithm combined QGA and simulated annealing (SA) algorithm [22] to optimize SVM parameters, tested the classification model based upon pancreatic images, and achieved better and stable accuracy.

### 2.2. Fruit Fly Optimization Algorithm

**Figure 1:**
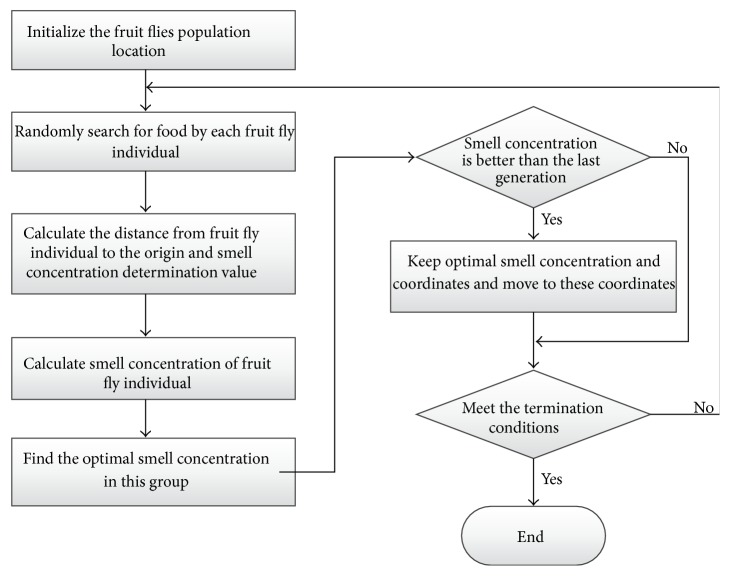
The flowchart of FOA.

Fruit fly optimization algorithm (FOA) [15] is a new method based on fruit fly foraging behavior for global optimization. The flowchart of FOA is shown in Figure 1.

The key steps of FOA are shown as follows.

**Step 1 .**

The position of population, (*X*_axis, *Y*_axis), is randomly initialized. *X*_axis and *Y*_axis are abscissa value and ordinate value of population's position, respectively.

**Step 2 .**

For each fruit fly, the direction and position of flying are randomly evaluated. It is represented as (9). (*X*_*i*_, *Y*_*i*_) is the new position of each fruit fly, where *i* ∈ [1, *M*]. *M* is the number of fruit flies in population:

(9) $$\begin{array}{c} X_{i}=X\_\text{axis}+\text{Random Value}\\ Y_{i}=Y\_\text{axis}+\text{Random Value}. \end{array}$$

**Step 3 .**

The distance (Dist_*i*_) from each fruit fly to the origin and the smell concentration determination value (*S*_*i*_) of each fruit fly are calculated as

(10) $$\begin{array}{c} {\text{Dist}}_{i}=\sqrt{X_{i}^{2}+Y_{i}^{2}}\\ S_{i}=\frac{1}{{\text{Dist}}_{i}}. \end{array}$$

**Step 4 .**

The smell concentration determination value is used in smell concentration determination function (fitness function) to calculate the smell concentration value as

(11) $$\begin{array}{c} {\text{Smell}}_{i}=\text{Function}\left(S_{i}\right). \end{array}$$

**Step 5 .**

The fruit fly which has the best smell concentration is found in population:

(12) $$\begin{array}{c} \left[\text{bestSmell bestIndex}\right]=\max\left(\text{Smell}\right). \end{array}$$

**Step 6 .**

The best smell concentration and its position (*x*, *y*) are saved. The fruit fly population moves to this position by vision.

**Step 7 .**

Step 2 to Step 5 are iterated. If the smell concentration is better than previous one, Step 6 is executed.

FOA is one of the intelligent optimization algorithms. It is easy to set up, easy to implement, and fast to optimize. But it also has some problems. In the phase of parameter initialization, FOA uses randomized strategy to determine initial point position. In the phase of fruit fly individual position update, blind search strategy is used. It is slow to converge and easy to fall into extreme values. At present, there are a number of evaluation criteria for classifier performance. In classifier optimization algorithms, classification accuracy and error rate are always used as the fitness function. But those criteria cannot reflect clinical prior knowledge. It is simply to evaluate an operating point and not strong enough when the distribution of class is changed.

## 3. Methodology

**Figure 2:**
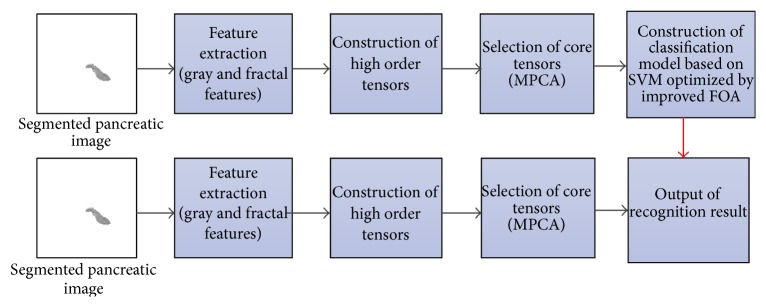
The flowchart of the proposed method.

The whole procedure of the proposed method is shown in Figure 2. The detailed process of the proposed method is as follows.

*(1) Feature Extraction*. We extract gray and fractal dimension features from the segmented pancreatic images, and then we normalize those features.

*(2) High Order Tensors Construction*. High order tensors are constructed based on the extracted features to represent pancreatic images.

*(3) Feature Selection*. In this paper we use the MPCA method to extract the eigen tensors for classification.

*(4) Pancreatic Cancer Classification*. After we obtain the eigen tensors by MPCA, we can treat the eigen tensors as input samples, and then we use the approach of SVM optimized by improved FOA to train classification model of pancreatic cancer image.

In the process, high order tensors construction and feature selection are carried out in accordance with [4]. So in this paper, we will no longer discuss them.

### 3.1. Improved FOA

Aiming at the existing problem of FOA, we introduce quantum to FOA and redefine a new fitness function as the smell concentration determination function.

#### 3.1.1. Quantum Fruit Fly Coding

In improved FOA (IFOA), quantum phase is used to code fruit flies' position. Compared with FOA which has the same number of fruit flies, the solution search space of quantum fruit flies is the double of the original fruit flies. The quantum fruit flies population position is shown as (13). When initializing, the quantum bit phase angle is *θ* = *π* · (2 · rand − 1), where rand ∈ [0,1], *j* ∈ [1, *n*], and *n* is the dimension of optimization problem. In this paper, *n* = 2:

(13) $$\begin{array}{c} X\_\text{axis}=\left[\theta_{x1},\theta_{x2},\ldots ,\theta_{xj}\right]\\ Y\_\text{axis}=\left[\theta_{y1},\theta_{y2},\ldots ,\theta_{yj}\right]. \end{array}$$

#### 3.1.2. Quantum Fruit Fly Smell Concentration Determination Value

As quantum phase is used to code fruit flies' position, each fruit fly has two solutions, namely, the cosine solution and the sine solution. The distance (Dist_*j*_^(*i*)^) from the *i*th fruit fly to the origin and the smell concentration determination value (*S*_*j*_^(*i*)^) of the *i*th fruit fly can be calculated as

(14) $$\begin{array}{c} {\text{Dist}}_{j}^{\left(i\right)}=\left[\begin{array}{c} \sqrt{{cos}^{2}\theta_{xj}^{\left(i\right)}+{cos}^{2}\theta_{yj}^{\left(i\right)}}\\ \sqrt{{sin}^{2}\theta_{xj}^{\left(i\right)}+{sin}^{2}\theta_{yj}^{\left(i\right)}} \end{array}\right] \end{array}$$

(15) $$\begin{array}{c} S_{j}^{\left(i\right)}=\frac{{\text{Dist}}_{j}^{\left(i\right)}}{\sqrt{2}}, \end{array}$$

where *i* ∈ [1, *M*], and *M* is the number of the fruit flies' populations. In (15), Dist is normalized to [0,1] and then assigned to *S*. The reason is to facilitate parameters zooming for optimizing SVM.

#### 3.1.3. Quantum Fruit Fly Smell Concentration Determination Function

False negative rate (FNR) is known as the rate of missed diagnosis. It is the percentage of actual sickness while identified as disease-free. FNR is complementary with the actual diagnostic sensitivity. False positive rate (FPR) is known as the misdiagnosis rate. It is the percentage of the actual disease-free while identified as sickness. FPR is complementary with the actual diagnostic specificity. In the process of actual disease diagnosis, if diagnosis with high sensitivity is used, the higher is the sensitivity, the less is the rate of missed diagnosis. That is to say, FNR is low. When diagnosis with high specificity is used, the misdiagnosis rate is low. That is to say, FPR is low. Therefore, in improved FOA, the mean of weighted sum of FNR and FPR in *k*-fold cross-validation is used as the smell concentration determination function. It is shown as

(16) $$\begin{array}{c} \text{Fitness}=\frac{1}{K}\overset{K}{\underset{k=1}{\sum }}\left[w\cdot \text{FNR}+\left(1-w\right)\cdot \text{FPR}\right]. \end{array}$$

In (16), *K* is the parameter of *k*-fold cross-validation, and *w* is the weight of FNR. If a fruit fly has small smell concentration value, it is good.

#### 3.1.4. Quantum Fruit Fly Mutation Operation

Quantum* not* gate is used to randomly change quantum fruit flies' positions. It not only increases the diversity of the population, but also avoids precocity. The quantum* not* gate based on phase coding is shown as

(17) $$\begin{array}{c} \theta_{j}^{\left(i\right)}=\frac{\pi }{2}-\theta_{j}^{\left(i\right)}. \end{array}$$

The mutation probability of an individual fruit fly is *P*_*m*_. If *P*_*m*_ is greater than a random number within (0,1), the two probability amplitudes of *X*-coordinate or *Y*-coordinate of the individual fruit fly randomly selected will be exchanged by quantum* not* gate.

The acceptance probability of mutated new fruit fly position obeys the Boltzmann probability distribution. It is shown as

(18) $$\begin{array}{c} P\left(\theta^{\left(i\right)*}⟹\theta^{\left(i\right)}\right)=\left\{\begin{array}{cc} 1, & F\left(\theta^{\left(i\right)*}\right)<F\left(\theta^{\left(i\right)}\right)\\ P_{i}, & F\left(\theta^{\left(i\right)*}\right)\geq F\left(\theta^{\left(i\right)}\right) \end{array}\right.\\ P_{i}={\left(1+\exp\left[\frac{F\left(\theta^{\left(i\right)}\right)-F\left(\theta^{\left(i\right)*}\right)}{l}\right]\right)}^{-1}. \end{array}$$

In (18), *θ* ^(*i*)*∗*^ is the mutated new fruit fly position, *θ* ^(*i*)^ is the original fruit fly position, *F*(·) is the smell concentration determination function, and *l* is iterations. If *F*(*θ* ^(*i*)*∗*^) < *F*(*θ* ^(*i*)^), the new position will be accepted by probability 1. Otherwise, the new position will be accepted by probability *P*_*i*_.

### 3.2. Construction of SVM Classifier Based on Improved FOA

**Figure 3:**
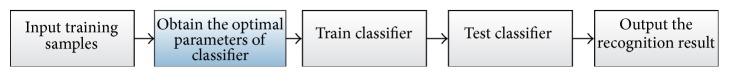
The framework of classifier construction.

**Figure 4:**
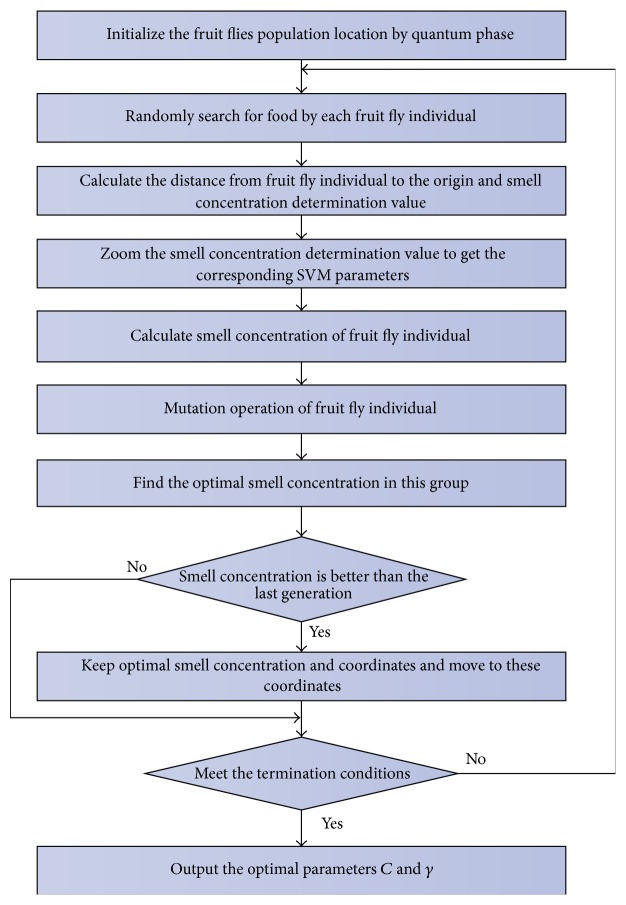
The flowchart of SVM parameter optimization based on improved FOA.

The framework of classifier construction and the flowchart of SVM parameter optimization based on improved FOA are shown in Figures 3 and 4, respectively. The process of classifier construction consists 3 steps, namely, obtaining classifier parameters, training classifier, and testing classifier.

The parameters of SVM, penalty factor *C*, and RBF kernel function parameter *γ* have great influence on the performance of classifier. *C* determines the promotion ability of SVM. The small value of *C* represents the penalty of empirical error being small, which can lead to “underfitting study.” The large value of *C* represents the penalty of empirical error being large, which can lead to “overfitting study.” The optimal value of *C* is different according to different data subspace, and selecting the optimal value of *C* can make the promotion ability better. SVM can map the input data of low dimensional space into high dimensional space by the kernel function. Vapnik [14] has found that the parameters of kernel function and penalty factor *C* have great influence on the performance of SVM. So the selection of parameters of penalty factor *C* and RBF kernel function parameter *γ* is important.

The detailed process for optimizing SVM parameters, penalty factor *C*, and RBF kernel function parameter *γ* is as follows.

**Step 1 .**

The population position (*X*_axis, *Y*_axis) is initialized by (13).

**Step 2 .**

For each fruit fly, the position and the direction of flying are randomly evaluated. It is shown as

(19) $$\begin{array}{c} X_{i}=X\_\text{axis}+V_{x}^{\left(i\right)}\\ Y_{i}=Y\_\text{axis}+V_{y}^{\left(i\right)}. \end{array}$$

In (19), *V* ∈ [−1,1], *i* ∈ [1, *M*], and *M* is the number of individuals in population.

**Step 3 .**

The distance from each fruit fly to the origin and the smell concentration determination value is calculated as (14) and (15).

**Step 4 .**

The smell concentration determination value is zoomed to get *C* and *γ*. It is shown as (20). *Cm* and *gm* are zoom multiples of *C* and *γ*, which can be obtained by prior knowledge:

(20) $$\begin{array}{c} C=Cm\cdot S_{1}^{\left(i\right)}\\ \gamma =gm\cdot S_{2}^{\left(i\right)}. \end{array}$$

**Step 5 .**

The smell concentration is calculated by (16). We set *K* = 5 and will discuss the value of *w* in the next section.

**Step 6 .**

If the individual fruit fly meets the mutation condition, the mutation operation will be done as (17) and (18).

**Step 7 .**

The fruit fly which has the best smell concentration is found as (21). bestSmell is the best smell concentration, bestIndex is the individual fruit fly which has the best smell concentration, and bestPos is the position of best smell concentration of the individual fruit fly:

(21) $$\begin{array}{c} \left[\begin{array}{c} \text{bestSmell}\\ \text{bestIndex}\\ \text{bestPos} \end{array}\right]=\min\left(\text{Smell}\right). \end{array}$$

**Step 8 .**

The axes and position of the best smell concentration are saved. The fruit fly population moves to this position by vision. It is shown as

(22) $$\begin{array}{c} \text{SmellBest}=\text{bestSmell} \end{array}$$

(23) $$\begin{array}{c} \text{Posbest}=\text{bestPos} \end{array}$$

(24) $$\begin{array}{c} X\_\text{axis}=X\left(\text{bestIndex}\right) \end{array}$$

(25) $$\begin{array}{c} Y\_\text{axis}=Y\left(\text{bestIndex}\right). \end{array}$$

**Step 9 .**

Step 2~Step 7 are iterated. If the smell concentration is better than previous one, Step 8 is executed. If the termination condition is satisfied, the optimum parameters will be returned.

## 4. Results and Discussion

### 4.1. Experimental Data

In this paper, abdominal CT images are used in experiments, which are provided by the radiology department of a hospital in Shenyang, China. Their resolution is 512 × 512 pixels, the scan slice thickness is 2 mm, and the format is DICOM. For the purpose of algorithm simulation, the DICOM image is transformed into BMP image. The grayscale is 256 and the resolution is 128 × 128. The detailed information of dataset is shown in Table 1.

### 4.2. Evaluation Criteria

According to the hybrid matrix, which is shown in Table 2, the evaluation criteria are calculated. In this paper, evaluation criteria consist of False Positive Rate (FPR), False Negative Rate (FNR), Accuracy, Precision,* F*1 value, and the running time of the algorithms. The mean square errors of evaluation criteria in many experiments are also used to evaluate the stability of the algorithm:

(26) $$\begin{array}{c} \text{FPR}=\frac{\text{FP}}{\text{N}}\\ \text{FNR}=\frac{\text{FN}}{\text{P}}\\ \text{Accuracy}=\frac{\text{TP}+\text{TN}}{\text{P}+\text{N}}\\ \text{Precision}=\frac{\text{TP}}{{\text{P}}^{\prime }}\\ F1=\frac{2}{1/\text{Precision}+1/\text{Recall}}. \end{array}$$

### 4.3. Prior Knowledge

**Figure 5:**
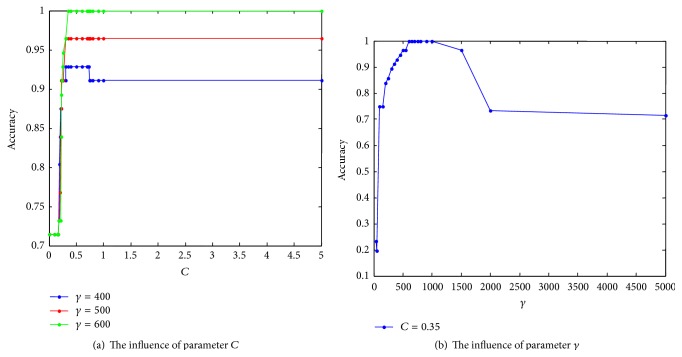
The effect of parameters *C* and *γ* on the classification accuracy.

Because of the sensitivity of initial scope for parameters optimization, we make the statistical analysis for the penalty factor *C* and RBF kernel function parameter *γ*, which obtains the prior knowledge of the parameters. The result is shown as in Figure 5.

From Figure 5, we can obtain the initial scope of *C* and *γ* by QSA that is [0.1,1] and [50,2000], respectively. And the scope of optimal solution is [0.3,0.5] and [500,1500], respectively. The scaling of *C* and *γ* is 1 and 2000, respectively.

### 4.4. Determination of FNR Weight

In an actual treatment, a patient was ill, but he was diagnosed as disease-free; then the treatment progress would be delayed and the cure opportunity would be reduced. On the contrary, if a patient was disease-free and was diagnosed with illness, patient would undergo further examination to make up the mistake. Therefore, it is believed that FNR is more important than FPR. The weight of FNR should be greater than FPR; that is to say, 0.5 ≤ *w* ≤ 1.

For different values of *w*, the experiment was run 10 times. Then we compared the mean value of evaluation criterions and their mean square error.

**Figure 6:**
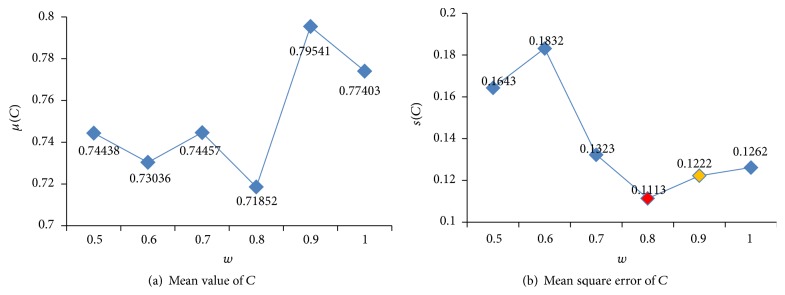
The status of parameter *C* for different *w*.

**Figure 7:**
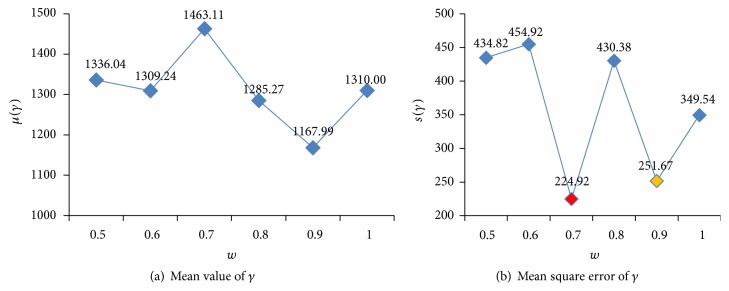
The status of parameter *γ* for different *w*.

**Figure 8:**
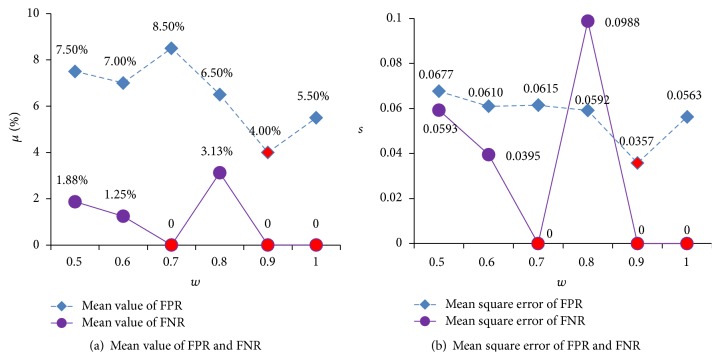
FPR and FNR status for different *w*.

**Figure 9:**
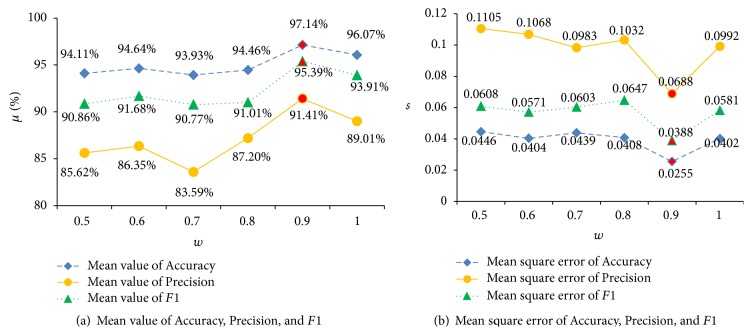
Accuracy, Precision, and *F*1 status for different *w*.

Figures 6 and 7 show the states of optimized parameters *C* and *γ* using different *w*.  Figure 8 illustrates the states of FNR and FPR.  Figure 9 demonstrates the states of Accuracy, Precision, and *F*1.

From Figures 6 and 7, it can be seen that the optimized parameters are found to be in line with prior knowledge. According to the mean square error of *C*, when *w* = 0.8, the algorithm is the most stable, and *w* = 0.9 is the second. According to the mean square error of *γ*, when *w* = 0.7, the algorithm is the most stable, and *w* = 0.9 is the second.

The better performance of the algorithm comes when the values of FNR and FPR are smaller. From Figure 8, for FPR, when *w* is 0.9, its mean value and mean square error are the smallest; for FNR, when *w* is 0.7, 0.9, or 1, its mean value and mean square error are the smallest. Greater values of Accuracy, Precision, and *F*1 can lead to better performance of the algorithm. From Figure 8, when *w* = 0.9, its mean values of Accuracy, Precision, and *F*1 are the greatest, and the mean square error is the smallest. Therefore, the final value of *w* is determined as 0.9.

### 4.5. Experimental Results and Analysis

Ten experiments of SVM optimized by improved FOA (IFOA-SVM) are randomly done. The experimental result is shown in Table 3.

From Table 3, it is known that the mean values of *C* and *γ* are 0.79541 and 1167.99183, respectively. The average of FPR is 4%, FNR is 0, Accuracy is 97.14%, Precision is 91.41%, *F*1 is 95.39%, and the time is 31.197 s.

**Figure 10:**
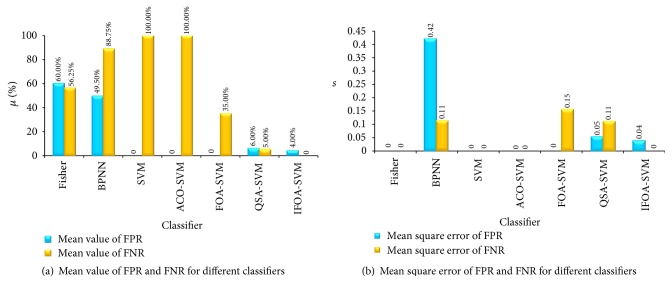
FPR and FNR status for different classifiers.

**Figure 11:**
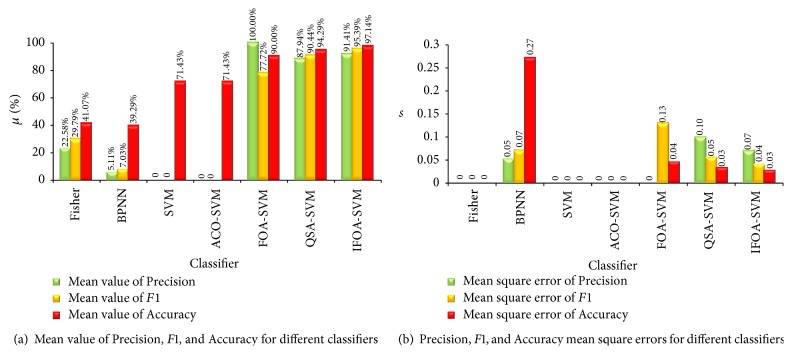
Accuracy, Precision, and *F*1 status for different classifiers.

**Figure 12:**
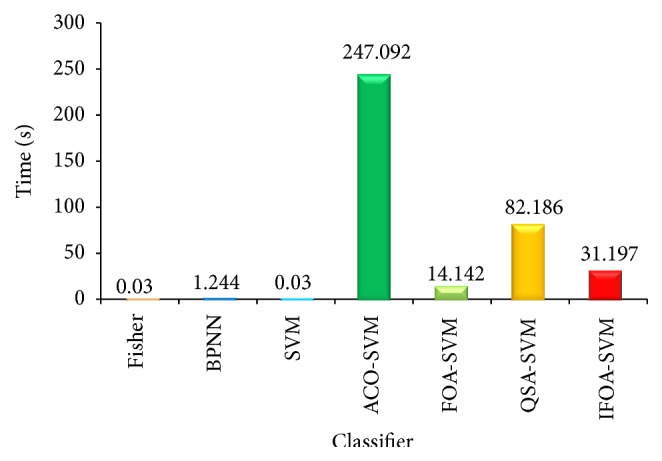
Running time for different classifiers.

Compared with other classifiers, the performance of IFOA-SVM is better as shown in Figures 10 and 11. In Figure 12, the comparison of running time is shown. Classifier Fisher is the Fisher linear classifier, classifier BPNN is the BP neural network, SVM is the common SVM, and ACO-SVM, FOA-SVM, and QSA-SVM are the optimized classifier SVM using ant colony algorithm, fruit fly optimal algorithm, and quantum simulated annealing, respectively. IFOA-SVM is the proposed method.

In Figure 10, FNR achieved 100% and FPR is 0 by SVM and ACO-SVM; that is to say, all patients are diagnosed free from diseases. This situation is not allowed in actual diagnosis. FNR of BPNN and Fisher are 88.75% and 56.25%, and FPR are 60% and 49.5%. So BPNN and Fisher lack credibility. FPR and FNR of FOA-SVM are 0 and 35%. It sometimes occurs in missed diagnosis situation. FPR and FNR of QSA-SVM are 6% and 5%. It might occur in missed diagnosis to few patients. FPR and FNR of the proposed IFOA-SVM are 4% and 0. It is better than other methods. IFOA-SVM achieves the best sensibility and stability.

In Figure 11, for the average of Precision, FOA-SVM is the best, which is 100%. IFOA-SVM takes the second place, which is 91.41%. And from Figure 11(b), FOA-SVM is the most stable. For the average of* F*1, the proposed IFOA-SVM achieves 95.39%, which is optimal. QSA-SVM is 90.44%, which takes the second place. But IFOA-SVM is more stable than QSA-SVM. For the average of Accuracy, IFOA-SVM is 97.14%, which is the best. QSA-SVM is 94.29%. FOA-SVM is 90%. SVM and ACO-SVM are 71.43%. Fisher and BPNN are less than 50%. Compared with mean square error of Accuracy, IFOA-SVM is the most stable.

In Figure 12, Fisher and SVM cost the least time, which is 0.03 s. BPNN is 1.244 s. FOA-SVM is 14.142 s. IFOA-SVM is 31.197 s. QSA-SVM is 82.186 s. ACO-SVM cost the most time, which is 247.092 s. In actual diagnosis, less time is better. The proposed IFOA-SVM is not the best but is not out of the way.

**Figure 13:**
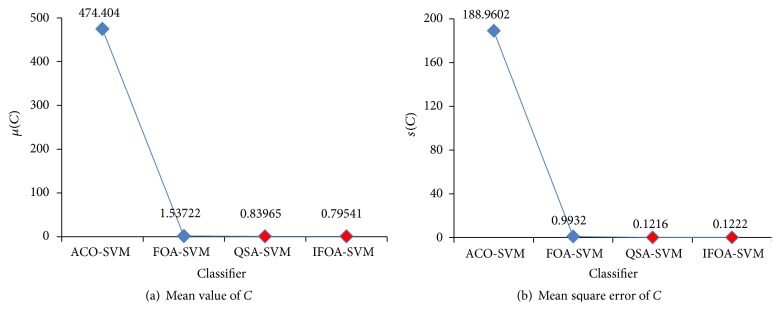
Parameter *C* status for different method.

**Figure 14:**
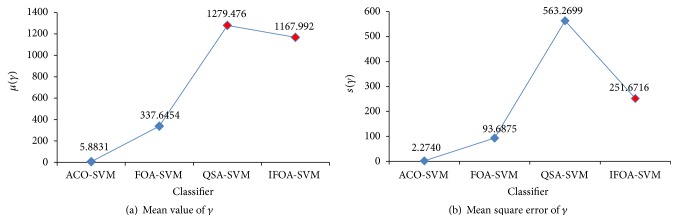
Parameter *γ* status for different method.

ACO-SVM, FOA-SVM, QSA-SVM, and the proposed IFOA-SVM can be used to optimize SVM parameters. In Figures 13 and 14, comparative results of mean value and mean square error of optimal parameters, *C* and *γ*, are shown based on those methods.

In the pancreatic cancer classifier based on ACO-SVM, *C* is oversize and *γ* is undersize. By using FOA-SVM, optimal parameters are not in estimation interval of prior knowledge, but in terms of mean square error of optimal parameters FOA is stable. When QSA-SVM and IFOA-SVM are used to optimize SVM parameters, optimal parameters are in estimation interval, and the stability of two methods is similar from mean square error of *C*. And in terms of mean square error of *γ*, IFOA-SVM is more stable than QSA-SVM.

## 5. Conclusion

In this paper, we introduced the concept of quantum to FOA to improve it. A new smell concentration determination function was defined in the improved FOA. The improved FOA was used to optimize the parameters of SVM and a classifier was constructed based on the optimized SVM. As an application, pancreatic cancer classifier was established. The proposed method achieved better classification performance. The first reason is that quantum coding and quantum operation increased the diversity of the population and avoided precocity. The second reason is that the redefined smell concentration determination function was more suitable to the actual diagnosis requirements. The third reason is the advantages of FOA which are easy to set up, easy to implement, and fast to optimize. Therefore, the proposed method can improve the classification performance of pancreatic cancer images and then assist doctors in diagnosing diseases.

## Figures and Tables

**Table 1:** Experimental data.

	Training samples	Testing samples	Total
Pancreatic cancer images (positive)	17	16	33

Normal images (negative)	41	40	81

Total	58	56	

**Table 2:** Hybrid matrix.

	Predicted positive example (P′)	Predicted negative example (N′)
Practical positive example (P)	True positive example (TP)	False negative example (FN)

Practical negative example (N)	False positive example (FP)	True negative example (TN)

**Table 3:** Experimental results of IFOA-SVM.

	C	*γ*	FPR	FNR	Accuracy	Precision	*F*1	Time (s)
1	0.8666	878.79	0	0	100.00%	100.00%	100.00%	31.78
2	0.7089	1003.4	2.50%	0	98.21%	94.12%	96.97%	31.64
3	0.7015	952.7641	2.50%	0	98.21%	94.12%	96.97%	31.35
4	0.8605	1401.3	5.00%	0	96.43%	88.89%	94.12%	30.49
5	0.9922	1314	5.00%	0	96.43%	88.89%	94.12%	31.09
6	0.8319	1364.5	5.00%	0	96.43%	88.89%	94.12%	31.28
7	0.6345	1050.6	2.50%	0	98.21%	94.12%	96.97%	30.7
8	0.9405	820.0642	0	0	100.00%	100.00%	100.00%	30.9
9	0.7671	1384.7	5.00%	0	96.43%	88.89%	94.12%	31.06
10	0.6504	1509.8	12.50%	0	91.07%	76.19%	86.49%	31.68
*μ*	0.79541	1167.99183	4.00%	0	97.14%	91.41%	95.39%	31.197
*s*	0.1222	251.6716	0.0357	0	0.0255	0.0688	0.0388	
